# *Candida auris* Candidemia in Kuwait, 2014

**DOI:** 10.3201/eid2106.150270

**Published:** 2015-06

**Authors:** Maha Emara, Suhail Ahmad, Ziauddin Khan, Leena Joseph, Ina’m Al-Obaid, Prashant Purohit, Ritu Bafna

**Affiliations:** Al-Sabah Hospital, Shuwaikh, Kuwait (M. Emara, I. Al-Obaid, P. Purohit, R. Bafna);; Faculty of Medicine, Kuwait University, Jabriyah, Kuwait (S. Ahmad, Z. Khan, L. Joseph)

**Keywords:** Candida auris, candidemia, Kuwait, Middle East, fungi, nosocomial, bloodstream pathogen

**To the Editor:** Recent reports from Asia (*1*–*4*) have highlighted the increasing incidence of the fungus *Candida auris* as a nosocomial bloodstream pathogen affecting persons of all age groups. We report a case of *C. auris* candidemia in a 27-year-old woman in Kuwait with a long history of chronic renal failure. On May 9, 2014, the patient was admitted to the intensive care unit with symptoms of septic shock secondary to lobar pneumonia and complicated by acute renal failure. The patient was known to have immotile cilia syndrome (primary ciliary dyskinesia) and bronchiectasis with recurrent episodes of sinusitis. Beginning on day 1, she received treatment with different courses of a wide range of broad-spectrum antimicrobial drugs. However, despite treatment, the patient’s condition continued to deteriorate. On day 12 after admission, a blood culture yielded yeast growth that was identified with 99% probability as *C. haemulonii* by using the Vitek 2 yeast identification system (bioMérieux, Marcy l’Etoile, France). As part of routine patient care, we sent the isolate (Kw1732/14) to the Mycology Reference Laboratory at Kuwait University for further identification and antifungal susceptibility testing. The isolate was resistant to fluconazole (MIC of >256 μg/mL), but it appeared susceptible to amphotericin B (MIC of 0.064 μg/mL), voriconazole (MIC of 0.38 μg/mL), and caspofungin (MIC of 0.064 μg/mL) by using the Etest (bioMérieux, Marcy l’Etoile, France). The patient was started on liposomal amphotericin B (150 mg/day), but the next day, she died from multiorgan failure.

On MAST ID CHROMagar Candida medium (Mast Group Ltd., Bootle, UK), the isolate formed pink colonies, which grew well at 42°C but not at 45°C. The isolate did not grow on BBL Mycosel Agar (BD, Sparks, MD, USA) containing 0.4 g cycloheximide per liter of medium. As with *C. auris* isolates from India and South Africa, this isolate assimilated *N*-acetyl glucosamine (*2*,*5*). Because the isolate showed reduced susceptibility to fluconazole, it was further characterized by sequencing of internal transcribed spacer and D1/D2 domains of ribosomal DNA. Genomic sequences for the internal transcribed spacer and D1/D2 regions (EMBL accession nos. LN624638 and LN626311) shared 99%–100% identity with sequences for corresponding regions of several *C. auris* strains (identification nos. CBS12874, CBS12875, CBS12876, CBS12880, CBS12882, CBS12886, and CBS12887, and several isolates from India).

*C. auris* was isolated in 2009 from the ear canal of a woman in Japan (*6*). The species has attracted attention because of its reduced susceptibility to azoles and amphotericin B (*2*,*5*) and its misidentification as *C. haemulonii* or *Rhodotorula glutinis* by commercial yeast identification systems (*1*,*4*). Because there are no reliable phenotypic methods for the rapid identification of *C. auris* and because molecular methods are not yet widely available, it is reasonable to infer that *C. auris* may be a more frequent cause of candidemia than previously recognized, particularly in Asian countries. A recently published multicenter study from India supports this view (*7*). In that study, a significantly higher occurrence of *C. auris* candidemia was reported among patients admitted in public sector hospitals compared with those in private hospitals (8.2 vs. 3.9%; p = 0.008) (*7*). The report reinforces the growing clinical implications of rare *Candida* spp. in the etiology of candidemia and highlights the role of molecular methods for their unequivocal identification.

## References

[1] Lee WG, Shin JH, Uh Y, Kang MG, Kim SH, Park KH, First three reported cases of nosocomial fungemia caused by *Candida auris.* J Clin Microbiol. 2011;49:3139–42 . 10.1128/JCM.00319-11 21715586 PMC3165631

[2] Chowdhary A, Anil Kumar V, Sharma C, Prakash A, Agarwal K, Babu R, Multidrug-resistant endemic clonal strain of *Candida auris* in India. Eur J Clin Microbiol Infect Dis. 2014;33:919–26. 10.1007/s10096-013-2027-1 24357342

[3] Chowdhary A, Sharma C, Duggal S, Agarwal K, Prakash A, Singh PK, New clonal strain of *Candida auris*, Delhi, India. Emerg Infect Dis. 2013;19:1670–3. 10.3201/eid1910.130393 24048006 PMC3810747

[4] Sarma S, Kumar N, Sharma S, Govil D, Ali T, Mehta Y, Candidemia caused by amphotericin B and fluconazole resistant *Candida auris.* Indian J Med Microbiol. 2013;31:90–1. 10.4103/0255-0857.108746 23508441

[5] Magobo RE, Corcoran C, Seetharam S, Govender NP. *Candida auris*–associated candidemia, South Africa. Emerg Infect Dis. 2014;20:1250–1. 10.3201/eid2007.131765 24963796 PMC4073876

[6] Satoh K, Makimura K, Hasumi Y, Nishiyama Y, Uchida K, Yamaguchi H. *Candida auris* sp. nov., a novel ascomycetous yeast isolated from the external ear canal of an inpatient in a Japanese hospital. Microbiol Immunol. 2009;53:41–4. 10.1111/j.1348-0421.2008.00083.x 19161556

[7] Chakrabarti A, Sood P, Rudramurthy SM, Chen S, Kaur H, Capoor M, Incidence, characteristics and outcome of ICU-acquired candidemia in India. Intensive Care Med. 2015;41:285–95. 10.1007/s00134-014-3603-2 25510301

